# Inter-and Intra-Examiner Reliability of Supraspinatus Muscle Tendon Palpation: A Cross-Sectional Study by Ultrasonography

**DOI:** 10.3390/medicina56020083

**Published:** 2020-02-18

**Authors:** Fermin Naranjo-Cinto, Giezi Falcon-Machado, Alejandro Garrido-Marin, Francisco Jose Senin-Camargo, Maria Amalia Jacome-Pumar, Ruben Fernandez-Matias, Daniel Pecos-Martin, Tomas Gallego-Izquierdo

**Affiliations:** 1 Physiotherapy and Pain Group, Department of Physical Therapy, University of Alcala, Alcalá de Henares, 28871 Madrid, Spain; ft.ferminnaranjo@hotmail.com (F.N.-C.); tomas.gallego@uah.es (T.G.-I.); 2 Instituto de Investigación Fisioterapia y Dolor, University of Alcala, Alcalá de Henares, 28805 Madrid, Spain; Giezi_92@hotmail.com (G.F.-M.); ruben.fernanmat@gmail.com (R.F.-M.); 3 CARMASALUD Clinical and Research Center, 28037 Madrid, Spain; alejandro.garrido@carmasalud.com; 4 Department of Physical Therapy and Podiatry, Faculty of Health, Exercise and Sport, European University of Madrid, 28670 Madrid, Spain; 5 Department of Biomedical Sciences, Medicine and Physiotherapy, Faculty of Physiotherapy, Universidade da Coruña, 15006 A Coruña, Spain; francisco.senin@udc.es; 6 MODES group, CITIC, Department of Mathematics, Faculty of Sciences, Universidade da Coruña, 15008 A Coruña, Spain; majacome@udc.es

**Keywords:** shoulder, rotator cuff, ultrasound imaging, palpation, reliability

## Abstract

*Background and objectives*: Palpation is an inherent and basic skill of health professionals—particularly manual therapists who base their diagnosis and treatment in a clinical environment. Many authors have previously described multiple palpation positions for supraspinatus muscle tendon (SMT); however, there are no current studies that evaluate palpation concordance reliability for the SMT in this particular position. This study aimed to investigate the intra- and inter-rater reliabilities of supraspinatus muscle tendon (SMT) palpation. *Materials and Methods*: Thirty-six healthy participants (14 females; aged 22–35 years) were recruited. Musculoskeletal ultrasound was used to measure the SMT localization after two physiotherapists performed the SMT palpation at two different times. The distance between the two marked points was used to analyze the analysis of true agreement between examiners. Finally, we analyzed if the demographic variables influenced the palpation procedure. *Results*: The intra-examiner reliability showed a high percentage of concordance for examiner 1 (E.1) (first palpation procedure (P.1) = 91.7%: second palpation procedure (P.2) = 95.8%) and examiner 2 (E.2) (P.1 = 91.6%; P.2 = 97.2%) and high percentage of inter-palpation agreement for E.1 (87.5%) and E.2 (88.9%). The inter-examiner reliability showed a high total concordance for the right shoulder (E.1 = 94.4%; E.2 = 95.8%) and left shoulder (E.1 = 93.05%; E.2 = 95.05%). The agreement (%) according to both examiners was 93.05% for the right shoulder and 94.4% for the left shoulder. The agreement between both examiners and the ultrasound (% of true agreement) was 92.9% for the right shoulder and 92.8% for the left shoulder. A statistically significant association (*p* = 0.02) was found for weight regarding concordance reliability; this was not seen for dominant arm, age, gender, body mass index, height, and tendon depth (*p* > 0.05). *Conclusions:* The SMT palpation technique showed a high level of concordance and reproducibility.

## 1. Introduction

Palpation is an inherent and basic skill of health professionals, particularly manual therapists who base their diagnosis and treatment in a clinical environment [1]. Thus, palpation abilities are still critical in physical testing [2,3].

Manual therapists usually treat the supraspinatus muscle tendon (SMT) due to its known correlation with shoulder pain and tendinopathy of the rotator cuff [3,4,5]. The muscle route is usually one made by the supraspinatus muscle from the second third of the supraspinatus pit to the greater humerus tuberosity. Current approaches speak of an aponeurotic expansion that is inserted underneath and laterally to the long tendon of the biceps [6,7,8]. Many authors have previously described multiple palpation positions for SMT [9,10,11,12,13].

Nevertheless, one of the most common positioning is the one described by Cyriax, who used a palpation technique to position the arm in the medial maximum rotation, adduction, and light hyperextension. This position is achieved when positioning the forearm behind the back, leaving no space between the arm and the chest. Cyriax confirmed that the SMT is placed laterally and parallel to the bicipital slide in this position, and also anterior to the acromion [14]. This position shows a statistically significant difference (*p* < 0.001) regarding the depth of the tendon from the surface of the skin in comparison with a normal anatomical position [15]. Different authors make use of this positioning technique in order to locate the SMT, not only for palpation, but also for carrying out an ultrasound test [16,17,18]. However, there are no current studies that evaluate palpation concordance reliability for the SMT in this particular position.

The depth from the skin surface to the SMT does not depend on demographic variables, but body mass index (BMI) is moderately correlated. The same study showed that the thickness of the SMT varies according to anatomic location among subjects; however, the differences between both arms were insignificant [15].

Ultrasound is widely used to obtain images of the musculoskeletal tissue—it offers images in real time with the same sensitivity as magnetic resonance image (MRI). Nevertheless, the sensitivity and specificity of ultrasound depends on the training and experience of the operator [19,20].

The main objective of this study is to evaluate the intra- and inter-examiner reliability in the SMT palpation procedure in correlation with age, gender, physical activity, BMI, dominant arm, and depth from the surface of the skin to the tendon. Ultrasound corroboration was performed in the location of SMT.

## 2. Materials and Methods

### 2.1. Study Design

A cross sectional descriptive study was carried out to assess the reliability of the SMT palpation between June and December 2017. Four highly qualified physiotherapists together with an expert in physiotherapy and musculoskeletal ultrasound participated throughout the study. The study was developed following the Strengthening the Reporting of Observational Studies in Epidemiology (STROBE) guidelines [21]. The study was also approved by the Ethics Committee of Investigation and Animal Experiments (Code CEIM/HU/2017/05).

### 2.2. Examiners

For the inter-intra examiner concordance, 2 physiotherapists with 3 years of experience in shoulder injuries performed the SMT palpation. A third qualified physiotherapist carried out the distance measurement between the two points marked as SMT by two initial evaluators. A final physiotherapist, who was an expert in musculoskeletal ultrasound, performed the ultrasound exploration of the marked spots.

Before this study started, three 1-h training sessions began with the objective of reaching a consensus regarding the positioning of subjects during the palpation, as well as the measurement of the distance between points and the ultrasound exploration protocol of SMT.

### 2.3. Subjects

Thirty-six subjects were recruited. The subjects participated voluntarily and received an information sheet regarding the aim and procedure of the study as well as an informed written consent.

The inclusion criteria included being older than 18 years of age, having signed the written consent form, and being able to place both upper limbs in the palpation position. The exclusion criteria were to have gone through shoulder surgery or to have suffered a traumatic process in the last year, experiencing shoulder pain, mobility restriction, or inability to place arms in the palpation position.

### 2.4. Sample Size Calculation

We used Epidat 4.2 software (Conselleria de Sanidade, Xunta de Galicia, Galicia, Spain) to determine the sample size with a kappa value of 0.80, a lower 95% confidence interval (CI) of 0.60, and an upper CI of 1.00 according to the recommendations of Cadogan and Scholtes [22,23]; 50% positive ratings were assumed for both raters; 36 subjects were needed based on this calculation. We targeted 45, considering an estimated 25% losses and abandonment. However, the sample was *n* = 36 with no losses, and thus *n* = 45 was not reached.

### 2.5. Ultrasound Test

A physiotherapist, who was an expert in musculoskeletal ultrasound with more than 5 years of experience, performed the ultrasound corroboration of the points marked by two examiners. The ultrasound was made by a high quality diagnostic ultrasound Philips iU22 (Philips Healthcare Inc., Bothell, Washington, USA) with a linear transducer with range of 7–17.0 MHz (L 17-5 Broadband Linear Array, 38- mm footprint) with B-mode images.

**Figure 1:**
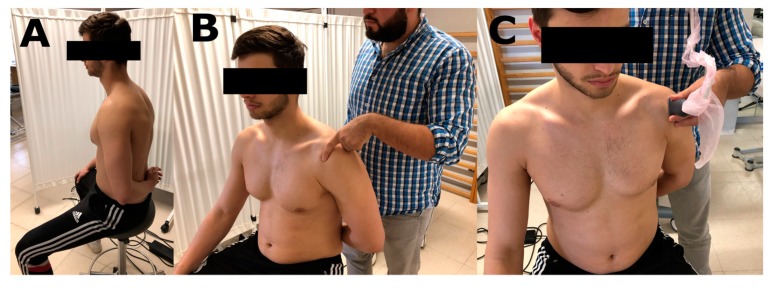
(**A**) Position of the subject during all procedures. (**B**) Supraspinatus muscle tendon palpation procedure. (**C**) Ultrasound evaluation procedure.

The ultrasound corroboration was performed in the same position in which the palpation was performed following the European Society of Musculoskeletal Radiology (ESMR) protocol for the determination of SMT. The measurement of the distance from the skin surface to the SMT was carried out using the greater tubercle of the humeral head as a bone reference point and tracing a 90° perpendicular line to the SMT. The image was frozen and the distance was measured (Figure 1) [15].

### 2.6. Procedure

For the palpation, signalling, distance measurement between points and ultrasound evaluation, the subject was seated on a backless bench with the arm in medial maximum rotation, adduction, and light hyperextension, respectively. This position was performed asking the subjects to place their forearm behind their lower back. Once the subjects were placed in the correct position, the examiners proceeded to the SMT palpation, considering bone references to the acromion and greater humeral tuberosity where the supraspinous tendon insertion should be found (Figure 1). This procedure was performed in both arms.

#### 2.6.1. Subjects Data Collection

The clinical record was used to acquire personal data including gender, dominant arm, age, BMI, weight, and height.

#### 2.6.2. Evaluation and Palpation Signaling

Once the subject was correctly placed (forearm behind the lower back), the examiners (E.1 and E.2) identified bony reliefs corresponding to the clavicle and acromion in order to later direct the palpation towards the greater humeral tubercle in search of the SMT [18,24]. The first examiner (E.1) was placed in an individual cabin with no contact with the rest of the examiners, and marked the SMT with an invisible and indelible ink marker for skin (Edding 8280) to hide the mark from the second evaluator. The second examiner (E. 2) was located in the exact same conditions as E.1 and marked the SMT with an indelible ink marker (Bic Marking 2300, permanent marker, green color). There was a time difference of 5 min between E.1 and E.2 to minimize the evidence of erythema or hyper sensibility in the area previously marked by E.1 [2]. Examiners E.1 and E.2 for SMT palpation were placed in the exact same position—behind and lateral to the shoulder that was going to be palpated. The palpation in both cases used the index finger with the right hand (both were right-handed).

#### 2.6.3. Evaluation and Palpation Points

The third examiner (E.3) used a Philips Lighting T8 18W ultraviolet light (Signify Inc., Amsterdam, Netherlands) and remarked on the signing made by E.1 with a permanent and indelible marker (Bic Marking 2300, permanent marker, red color) to make both points visible for correct ultrasound evaluation. After this procedure, the distance between both systems was measured with an absolute digimatic gauge (Mitutoyo Co., Kanagawa, Japan) to evaluate the inter-examiner true agreement.

To evaluate the true agreement, we used a cutoff point of 15 mm because it is considered to be the average of a fingerprint [25]. The true agreement was defined as a distance between both points less or equal to 15 mm, with the SMT palpation points of both examiners corresponding with the ultrasound results.

#### 2.6.4. Ultrasound Evaluation Made by an Expert Clinician

A fourth examiner (E.4) evaluated the ultrasound with both points as previously marked by E.1 and E.2 to determine if these matched with the ultrasound projection of the SMT.

#### 2.6.5. Second Evaluation and Randomization

Once every subject had been completely evaluated, a fifth examiner (E.5) deleted the marks of E.1 and E.3 with a piece of cotton and alcohol. Once all the ink had completely disappeared, the subjects were randomly asked to go through another evaluation with the help of EPIDAT 4.2. This second evaluation was made 30 min after the first one [26,27] and repeated the procedure. These 30 min avoided the patient recalling the first palpation. The examiner had no contact between the evaluations, ensuring independence between measurements and any type of interaction in which they could share results.

### 2.7. Statistical Analysis

We calculated the percentage (%) of concordance for each tester to establish intra-examiner reliability in each palpation procedure (E.4 through ultrasound corroborates that the marking on that point corresponds to a real SMT projection). The inter-palpation reliability of E.1 and E.2 was then measured based on percentage (%) of agreement and Cohen’s Kappa with different ranges of κ-values: poor (< 0.20), fair (0.20–0.40), moderate (0.41–0.60), good (0.61–0.80), and very good (0.81–1.00) reliability [28]. Moreover, a McNemar test was performed with continuity corrections in order to compare if there is a systematic difference among both palpations. Cohen’s Kappa is extremely sensitive and, in some cases, biased (difference prevalence between 1 = concordance and 0 = no concordance), Gwet’s Agreement Coefficient (AC1) was also calculated [29].

Furthermore, inter-examiner reliability was also measured by the percentage (%) of agreement as well as Cohen´s Kappa and Gwet’s AC1. McNemar´s test was used to study if there were systematic differences between examiners. The prevalence index, bias index, prevalence-adjusted-bias-adjusted-kappa (PABAK), and maximum kappa attainable (κ_max_) were also calculated [30].

Finally, the percentage (%) of true agreement was calculated to determine if there is concordance between both examiners (distance less or equal to 15 mm) and also concordance with the ultrasound. Finally, a Chi-squared test and logistic regression were carried out to determine if gender, dominant arm, age, BMI, weight, height, and SMT depth affected palpation.

Kappa and Gwet analyses were performed with statistical software R Version 3.5.3 (R Core Team 2019). (R is a language and environment for statistical computing (R Foundation for Statistical Computing, Vienna, Austria)). All other analyses were performed with SPSS 24.0 (Windows; SPSS IBM, Chicago, IL, USA). All analyses were conducted considering an α level of 0.05, with 95% confidence intervals (CI).

## 3. Results

### 3.1. Demographic Data

A total of 40 subjects were interviewed for the study; however, three of them did not meet the inclusion criteria, two had previous shoulder injuries in the past year, and one had limitations when placing the arm behind the back. Two other subjects did not turn up. Thus, 36 subjects (14 females, aged 22–35 years) were included. The demographic characteristics of the subjects are presented in Table 1.

### 3.2. Reliability and Intra-Examiner Concordance

The intra-examiner reliability (Table 2) showed a high percentage of concordance for E.1 (first palpation procedure (P.1) = 91.7%: second palpation procedure (P.2) = 95.8%) and E.2 (P.1 = 91.6%; P.2 = 97.2%), as well as a high percentage of inter-palpation agreement for E.1 (87.5%) and E.2 (88.9%).

### 3.3. Inter-Examiner Concordance Reliability

The inter-examiner reliability (Table 3) showed a high total concordance for the right shoulder (E.1 = 94.4%; E.2 = 95.8%) and left shoulder (E.1 = 93.05%; E.2 = 95.05%). The total percentage of agreement (%) according to both examiners was 93.05% for the right shoulder and 94.4% for the left shoulder.

### 3.4. Agreement Between Inter-Examiner Concordance and Ultrasound

In terms of concordance between both examiners (distance less or equal to 15 mm) and also concordance with the ultrasound, the real percentage of agreement between both examiners and the ultrasound (% of true agreement) was 92.9% for the right shoulder and 92.8% for the left shoulder (Table 4).

### 3.5. Effect of Palpation & Subjects Characteristics

**Figure 2:**
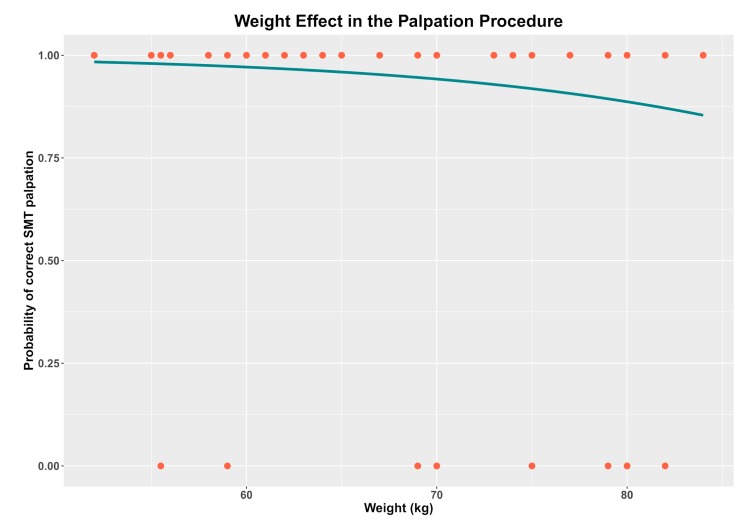
Effect of weight in the palpation of the supraspinatus muscle tendon (SMT). Red dots = observed concordance (1 = yes, 0 = no); blue line = predicted probability of correct SMT palpation.

Statistically significant effects were not found after SMT palpation for gender (*p* = 0.75), arm side (*p* = 0.45), dominance arm (*p* = 0.80), age (*p* = 0.95), tendon depth (*p* = 0.67), BMI (*p* = 0.19), or height (*p* = 0.10) (Table 5 and Table 6). Nevertheless, the effect of weight was shown to be statistically significant (*p* = 0.02) for the SMT palpation procedure (Figure 2). Although the effect was not statistically significant, better results were obtained in the second palpation procedure than the first one (*p* = 0.08) (Table 5).

## 4. Discussion

This is the first study to evaluate the reliability of the concordance and reproducibility of the palpation of the SMT. For intra-examiner concordance reliability, there was total concordance in both arms at 91.7% in the first palpation and 95.8% in the second palpation for E.1 and an agreement percentage of 87.5%. For E.2, a concordance percentage was obtained in P.1 with 91.6% and 97.2% for P.2; the agreement percentage was 88.9%. These results correlate with high intra-examiner reliability in the SMT palpation. These results can be compared with previous studies published regarding muscular and bone palpations [31,32].

To determine inter-examiner concordance agreement, a percentage (%) of agreement of 93.05% was obtained for the right arm and 94.4% for the left arm. The concordance (%) for the right arm in the first palpation was 94.4% and 95.8% for the second palpation; 93.05% was obtained in both palpations for the left arm.

Furthermore, differences in terms of SMT thickness and depth from the skin are small between both sides in the same subject [15]. This confirms that the subjects’ dominance is not a concordance-determinant factor in SMT palpation. The results for concordance reliability among examiners and the ultrasound results showed a total percentage (%) of true agreement of 92.9% for the right arm and 92.8% for the left one. The second palpation of the left arm had the higher percentage of true agreement with 97.1% and the first palpation of the left arm had the lower percentage of true agreement with 88.9%.

Sociodemographic variables such as gender, dominant arm, age, and BMI were evaluated. A statistically significant difference for these variables was not found regarding SMT location, which matches previous studies related to palpation and location of anatomic structures [33,34]. However, weight showed statistically significant differences, even if BMI did not (*p* =.02).

Mattlingly and Mackarey [35] determined that the position in which SMT showed greater exposure is in maximum shoulder adduction, maximum medial rotation, and maximum hypertension [35]. This position is different from the forearm behind the back position regarding the amount of extension performed. This may be directly proportional to SMT exposure. Different authors used this positioning technique for the SMT examination [36,37].

However, other groups currently use the position described by Cyriax as the main position for ultrasound examination and SMT palpation [17,18]. One goal here was to determine palpation concordance reliability of the SMT, and it only considered the forearm behind the back position and hyperextension degrees determined by the morphological characteristics of the subject´s thorax. This led to high agreement among examiners and the ultrasound image. This finding may determine that the forearm behind the back position was valid for an SMT palpation and that the palpation procedure was repeatable. Vosloo et al. [38] reaffirmed the fact that the rotator cuff—which is part of the SMT—is a complete unit. However, in terms of ultrasound, the image that corresponds to the SMT is accepted supporting the fact that SMT palpation can be validated via an ultrasound test [16].

### Limitations

The measurements of palpation concordance of the SMT were performed in a specific position in which the supraspinatus muscle sinewy part was exposed for palpation. However, this position may be symptomatic in subjects with previous SMT injury or mobility limitations; thus, this study cannot be extrapolated to symptomatic patients. An additional limitation of this study was not considering palpation perception of the evaluators. Thus, it is not possible to determine if the level of agreement is based on palpation concordance or in the SMT structure based on the anatomic references. This will be considered in future studies.

## 5. Conclusions

The results showed that SMT has high intra- and inter-examiner concordance reliability in asymptomatic subjects following Cyriax´s anatomic reference together with articular positioning techniques when palpation was performed. There was a high level of reproducibility in the palpation technique.

## Figures and Tables

**Table 1:** Characteristics of the patients (*n* = 36).

Characteristic	Mean (SD)	Range
Age, years	24.28 (2.89)	22–35
Weight, kg	68.07 (8.52)	52–84
Height, m	1.74 (0.09)	1.60–1.90
BMI, kg/m^2^	22.54 (1.86)	18.71–26.03
Tendon depth, mm		
Right	13.06 (3.54)	7.40–20.80
Left	12.14 (2.73)	6–17.70
Sex, *n* (%)		
Male	22 (61.1%)	
Female	14 (38.9%)	
Dominant side, *n* (%)		
Right	30 (83.3%)	
Left	6 (16.7%)	

Abbreviations: SD, standard deviation; BMI, body mass index.

**Table 2:** Intra-examiner reliability between both SMT palpation procedures.

Test (95% CI)	Examiner 1						Examiner 2					
	Right Arm		Left Arm		Total		Right Arm		Left Arm		Total	
	P.1	P.2	P.1	P.2	P.1	P.2	P.1	P.2	P.1	P.2	P.1	P.2
Concordance (%) ^*^	94.4%	94.4%	88.9%	97.2%	91.7%	95.8%	91.7%	100%	91.7%	94.4%	91.6%	97.2%
Agreement (%) ^†^	88.8%		86.1%		87.5%		91.7%		86.1%		88.9%	
Cohen’s Kappa (κ)	−0.06 (−0.39, 0.27)		−0.05 (−0.30, 0.21)		−0.06 (−0.37, 0.12)		0.00		−0.0 7(−0.39, 0.25)		−0.04 (−0.24, 0.15)	
Prevalence Index	−0.89 (−0.99, −0.79)		−0.86 (−0.97, −0.75)		−0.88 (−0.95, −0.80)		−0.92 (−1.00, −0.83)		−0.86 (−0.97, −0.75)		−0.89 (−0.96, −0.82)	
Bias Index	0.05 (−0.11, 0.11)		0.08 (−0.03, 0.20)		0.04 (−0.04, 0.12)		0.08 (−0.01, 0.17)		0.03 (−0.09, 0.15)		0.06 (−0.02, 0.13)	
PABAK	0.78 (0.48, 0.94)		0.72 (0.41, 0.91)		0.75 (0.55, 0.88)		0.83 (0.55, 0.96)		0.72 (0.41, 0.91)		0.78 (0.59, 0.90)	
κ_max_	1.00		0.37 (0.12, 0.63)		0.65 (0.43, 0.86)		0.00		0.79 (0.47, 1.00)		0.48 (0.28, 0.68)	
Gwet’s AC1	0.88 (0.74, 1.00)		0.84 (0.69, 0.99)		0.86 (0.76, 0.96)		0.91 (0.80, 1.00)		0.84 (0.69, 0.99)		0.88 (0.78, 0.97)	
McNemar Test	*p* = 1.00		*p* =0.37		*p* =0.51		*p* =0.25		*p* = 1.00		*p* =0.29	

Abbreviations: CI, confidence interval; P.1, first palpation procedure; P.2, second palpation procedure; SMT, supraspinatus muscle tendon; total, both arms; PABAK, prevalence-adjusted-bias-adjusted-kappa; κ_max_, maximum kappa attainable. ^*^ Concordance percentage with the ultrasound test. The fourth examiner used an ultrasound test to corroborate that the marked point corresponds to the real supraspinatus tendon projection. ^†^ Percentage of agreement among palpation results.

**Table 3:** Inter-examiner reliability of each SMT palpation.

Test (95% CI)	Right Arm						Left Arm					
	P.1		P.2		Total		P.1		P.2		Total	
	E.1	E.2	E.1	E.2	E.1	E.2	E.1	E.2	E.1	E.2	E.1	E.2
Concordance (%) *	94.4%	91.7%	94.4%	100%	94.4%	95.8%	88.9%	91.7%	97.2%	94.4%	93.05%	93.05%
Agreement (%) ^†^	91.7%		94.4%		93.05%		97.2%		91.7%		94.4%	
Cohen’s Kappa (κ)	0.36 (0.038, 0.68)		0.00		0.25 (0.02, 0.48)		0.84 (0.52, 1.00)		−0.04 (−0.34, 0.27)		0.57 (0.34, 0.80)	
Prevalence Index	−0.86 (−0.98, −0.74)		−0.94 (−1.00, −0.87)		−0.90 (−0.97, −0.83)		−0.81 (−0.94, −0.67)		−0.92 (−1.00, −0.83)		−0.86 (−0.94, −0.78)	
Bias Index	−0.03 (−0.14, 0.09)		0.06 (−0.02, 0.13)		0.01 (−0.06, 0.08)		0.03 (−0.11, 0.16)		−0.03 (−0.12, 0.06)		0.00 (−0.08, 0.08)	
PABAK	0.83 (0.55, 0.96)		0.89 (0.63, 0.99)		0.86 (0.69, 0.95)		0.94 (0.71, 1.00)		0.83 (0.55, 0.96)		0.89 (0.73, 0.97)	
κ_max_	0.79 (0.47, 1.00)		1.00		0.85 (0.62, 1.00)		0.84 (0.52, 1.00)		0.65 (0.35, 1.00)		1.00	
Gwet’s AC1	0.90 (0.79, 1.00)		0.94 (0.85, 1.00)		0.92 (0.85, 0.99)		0.97 (0.90, 1.00)		0.91 (0.80, 1.00)		0.94 (0.87, 1.00)	
McNemar	*p* = 1.00		*p* =0.48		*p* = 1.00		*p* = 1.00		*p* = 1.00		*p* = 1.00	

Abbreviations: CI, confidence interval; E.1, examiner 1; E.2, examiner 2; P.1, first palpation procedure; P.2, second palpation procedure; SMT, supraspinatus muscle tendon; total, both arms; PABAK, prevalence-adjusted-bias-adjusted-kappa; κ_max_, maximum kappa attainable. ^*^Concordance percentage with the ultrasound test. The fourth examiner corroborated that the point marked corresponds with the real supraspinatus tendon projection via ultrasound. ^†^ Percentage of agreement of times in which there was agreement between the results of both examiners.

**Table 4:** Percentage of concordance and agreement between both examiners and the ultrasound in the SMT location.

Test	Right Arm						Left Arm					
	P.1		P.2		Total		P.1		P.2		Total	
	E.1	E.2	E.1	E.2	E.1	E.2	E.1	E.2	E.1	E.2	E.1	E.2
Concordance (%) ^*^	94.4%	91.6%	94.4%	100%	94.4%	95.8%	88.8%	91.6%	97.2%	94.4%	93.05%	93.05%
Agreement (%)	91.6%		94.4%		93.05%		97.2%		91.6%		94.4%	
Distance ≤15mm (%) ^†^	97.2%		100%		98.6%		100%		94.4%		97.2%	
% true agreement ^‡^	91.4%		94.4%		92.9%		88.9%		97.1%		92.8%	

Abbreviations: E.1, examiner 1; E.2, examiner 2; P.1, first palpation procedure; P.2, second palpation procedure; SMT, supraspinatus muscle tendon; total, both arms. ^*^ Concordance percentage with the ultrasound test. The fourth examiner corroborated that the point marked corresponds with the real supraspinatus tendon projection via ultrasound. ^†^ Percentage of agreement of times in which there was agreement between results of both examiners. ^‡^Once determined as concordance between both examiners (distance ≤15 mm); percentage of times in which there also was concordance between ultrasound and both examiners.

**Table 5:** Concordance percentages based on sex, arm, and palpation.

Outcome	Concordance Percentage		*p*-Value
Gender	Men	Women	0.75
	93.8%	94.6%	
Arm side	Right	Left	0.45
	95.1%	93.1%	
Arm dominance	Dominant	Non-dominant	0.80
	94.4%	93.8%	
Palpation procedure	First	Second	0.08
	91.7%	96.5%	

**Table 6:** Subject characteristics and significance of their effects on SMT palpation concordance.

Characteristic	Concordance	Mean	SD	Min	1Q	Medium	3Q	Max.	*p-*Value
SMT depth, mm	Yes	12.58	3.19	6.0	10.3	12.4	14.3	20.5	0.67
	No	12.91	2.52	8.3	11.8	12.7	14.3	19.2	
Height, m	Yes	1.73	0.09	1.60	1.66	1.73	1.80	1.90	0.10
	No	1.77	0.08	1.64	1.74	1.80	1.85	1.88	
Weight, kg	Yes	67.78	8.32	52.0	61.0	67.0	75.0	84.0	0.02
	No	72.64	8.74	55.5	70.0	75.0	80.0	82.0	
BMI, kg/cm^2^	Yes	22.50	1.82	18.71	21.74	22.64	23.46	26.03	0.19
	No	23.10	2.03	19.49	22.64	23.12	24.69	26.03	
Age, years	Yes	24.28	2.93	22	23	23	25	35	0.95
	No	24.23	1.35	22	23	24	25	26	

Abbreviations: 1Q, first quartile; 3Q, third quartile; BMI, body mass index; Max, maximum; Min, minimum; SD, standard deviation; and SMT, supraspinatus muscle tendon.
